# The Total Antioxidant Status, Serum Selenium Concentrations and the Ultrasound Assessment Carotid Intima Media Thickness in Patients with Arterial Hypertension

**DOI:** 10.3390/antiox10010063

**Published:** 2021-01-06

**Authors:** Paweł Gać, Małgorzata Poręba, Lidia Januszewska, Adam Prokopowicz, Helena Martynowicz, Grzegorz Mazur, Rafał Poręba

**Affiliations:** 1 Department of Hygiene, Wroclaw Medical University, 50-368 Wroclaw, Poland; lidia.januszewska@umed.wroc.pl; 2 Centre for Diagnostic Imaging, 4th Military Hospital, 50-981 Wroclaw, Poland; 3 Department of Pathophysiology, Wroclaw Medical University, 50-368 Wroclaw, Poland; malgorzata.poreba@umed.wroc.pl; 4 Institute of Occupational Medicine and Environmental Health in Sosnowiec, 41-200 Sosnowiec, Poland; adam.prokopowicz@interia.com; 5 Department of Internal Medicine, Occupational Diseases and Hypertension, Wroclaw Medical University, 50-556 Wroclaw, Poland; helena.martynowicz@umed.wroc.pl (H.M.); gregorz.mazur@umed.wroc.pl (G.M.); rafal.poreba@umed.wroc.pl (R.P.)

**Keywords:** arterial hypertension, selenium, antioxidant status, carotid arteries, intima media thickness

## Abstract

The objective of the study was to establish the correlation between serum selenium concentrations, total antioxidant status, and the carotid intima media thickness in ultrasound assessment in patients with arterial hypertension. A group of 76 people suffering from arterial hypertension was qualified to participate in the study. The mean age of the respondents was 53.48 ± 12.78. Serum selenium concentrations (Se-S) and total antioxidant status (TAS) were determined in all respondents. Se-S were determined by hydride generation atomic absorption spectroscopy (HGAAS). The antioxidant status was assessed by the enzyme-linked immunosorbent assay (ELISA). In addition, an ultrasound exam of the carotid arteries was performed, and the intima media thickness (cIMT) was measured. In the study group, Se-S and TAS were 89.73 ± 18.99 µg/L and 1.18 ± 0.35 mM. However, the cIMT measured using ultrasound was 0.68 ± 0.15 mm. cIMT was significantly greater in patients with arterial hypertension with Se-S < median in comparison to patients with arterial hypertension with Se-S ≥ median (0.73 ± 0.19 mm vs. 0.65 ± 0.10 mm, *p* < 0.05), as well as in patients with arterial hypertension with TAS < median than in patients with arterial hypertension with TAS ≥ median (0.79 ± 0.18 mm vs. 0.56 ± 0.13 mm, *p* < 0.05). In regression analysis, older age, higher BMI, smoking, and lower serum selenium concentrations were independently correlated with the greater cIMT. Higher BMI and smoking were independent risk factors for the lower TAS, and the use of ACE inhibitors, β-blockers, and higher Se-S were independent factors of protection against the lower TAS. In patients with arterial hypertension, the lower total antioxidant status due to lower serum selenium concentrations may be correlated with an increase of the carotid intima media thickness measured using ultrasound.

## 1. Introduction

Selenium is an essential micronutrient that has a huge impact on human health. It is a component of many enzymes and it plays a key role in the optimization of metabolic reactions and protection against oxidative damage [1,2,3]. In humans, there are 25 genes for selenoproteins, many of which act as enzymes. They are necessary for the proper functioning of the brain, thyroid gland, and immune system, as well as for the preservation of fertility [4,5].

One of the aspects of scientific research on the importance of selenium is research determining its impact on the cardiovascular system, including the development and course of arterial hypertension [6]. The correlation of low blood selenium concentrations with the high blood pressure values in Keshan disease has undoubtedly been proven. It has been documented that the symptoms of the disease, including cardiac arrhythmias, cardiomyopathy, and arterial hypertension, resolve after selenium supplementation [7,8]. Studies conducted so far give contradictory results, some of them have not shown a correlation between blood selenium concentrations and arterial hypertension, while others have shown that such a correlation exists [9]. There is no agreement on the direction of the potential correlation. In several studies, lower blood selenium concentrations in patients with arterial hypertension have been observed but there are also reports of the opposite correlation [10,11,12,13].

Arterial hypertension is one of the major risk factors for cardiovascular disease. Arterial hypertension can occur on its own, but most often it is accompanied by other abnormalities, such as dyslipidemia and glucose intolerance, which increase its impact on the risk of cardiovascular diseases [14]. Consideration of the cardiovascular consequences of hypertension enables a more precise assessment of the risk of cardiovascular events. The subclinical evaluation of organ complications in hypertension focuses on abnormal pulse pressure, left ventricular hypertrophy, renal dysfunction (elevated serum creatinine, elevated albumin/creatinine ratio, microalbuminuria, decreased estimated glomerular filtration rate), and adverse vascular changes such as increased carotid intima media thickness (cIMT), lowering of the ankle brachial index (ABI), decreased flow-mediated dilatation (FMD) [15,16].

According to the recommendations of scientific societies, increased cIMT, like the presence of atherosclerotic plaques in the carotid arteries, is a recognized organ complication of arterial hypertension. cIMT thickening is an early marker of atherosclerosis, which is observed before the formation of atherosclerotic plaque. The normative range of cIMT is estimated to be within 0.4–0.9 mm, with an increment of about 0.1 mm for each decade of life [17]. Moreover, cIMT assessed by ultrasound has a prognostic significance. cIMT increased by only 0.1 mm significantly increases the risk of myocardial infarction and stroke [18]. Measurement of cIMT enables the prediction of the occurrence of stroke and myocardial infarction in a manner independent of traditional risk factors and the discussed correlation between cIMT and cardiovascular risk is linear [19].

In connection with the above-mentioned importance of selenium in the body (also to maintain the redox balance of the system), the postulated role of selenium in the development of hypertension, the importance of cardiovascular consequences of arterial hypertension as predictors of cardiovascular risks, and the diagnostic usefulness of ultrasound assessment of the carotid intima media thickness, it seems justified to verify the correlation between serum selenium concentrations, the total antioxidant status, and the carotid intima media thickness in ultrasound assessment in patients with arterial hypertension without other clinically evident cardiovascular diseases.

The objective of the study was to establish the correlation between serum selenium concentrations, the total antioxidant status, and the carotid intima media thickness in ultrasound assessment in patients with arterial hypertension.

## 2. Material and Methods

The studies were carried out as part of the research project entitled “Selenium and zinc deficiency as predictors of subclinical cardiovascular complications in hypertension” (Simple STM.A100.17.050), which was approved by the local ethics committee.

The studies were conducted between 2017–2019 in the hypertensiology clinic of the university hospital. The study group of patients was recruited from among those hospitalized in the clinic to evaluate the effectiveness and possible optimization of the treatment of arterial hypertension and to assess the cardiovascular consequences of the underlying disease. The inclusion criteria for the study were: age over 18 years, diagnosed and pharmacologically treated arterial hypertension, and no modification of the therapy of arterial hypertension in the last 12 months. Based on the adopted inclusion criteria, 141 patients were qualified to participate in the study. The size of the originally recruited group of respondents was reduced by excluding persons meeting the adopted exclusion criteria. Exclusion criteria were type 2 diabetes, ischemic heart disease, stroke, peripheral arterial disease, renal failure, and hyperthyroidism or hypothyroidism. Based on the accepted exclusion criteria, a total of 65 patients were excluded from the study: 44 people due to type 2 diabetes, 22—ischemic heart disease, 19—stroke, 15—peripheral arterial disease, 2—renal failure, and 2—hyperthyroidism or hypothyroidism. Most of the patients excluded from the study had more than one exclusion criterion. Ultimately, the study group consisted of 76 people. The clinical characteristics of the study group of patients are presented in Table 1.

The project methodology included a questionnaire, laboratory analysis, and an ultrasound exam of the carotid arteries.

The questionnaire included questions about the patient’s health (especially treatment and management of arterial hypertension), the presence of comorbidities, and the basic risk factors for cardiovascular diseases. The performed laboratory analyses included the determination of the basic parameters of lipid and carbohydrate metabolism in the blood, determination of serum selenium concentrations (Se-S), and determination of serum antioxidant status (TAS).

Total cholesterol, LDL and HDL, triglycerides, and blood glucose were determined using commercially available standard tests.

Serum selenium concentrations were determined using hydride generation atomic absorption spectroscopy (HGAAS). The antioxidant status (TAS) was assessed using the enzyme-linked immunosorbent assay (ELISA) according to the manufacturer’s instructions (Cayman Chemical, catalog no. 709001). The Se-S and TAS determination methodology used was analogous to that described previously, for example, by Gać et al. (2020) [20].

Carotid ultrasound exams were performed using the ALOKA ProSound 6 device (Aloka Inc., Tokyo, Japan). The exams were performed for the presence of (significant/ insignificant) carotid stenosis and/or occlusion. In addition, the intima media thickness (cIMT) was assessed. The exams were performed in the supine position with the patient’s head tilted about 30–45° from the examined artery. A linear probe with a frequency of 7–10 MHz was used for the measurements. The exams were begun with B-mode imaging, identifying the common carotid artery (CCA) in the transverse plane. In the longitudinal plane, the vessel was visualized using the optimal depth and setting the focus (single) to the depth of the artery being assessed. The IMT measurements were taken 1–2 cm below the division for the internal carotid (ICA) and external carotid (ECA) artery, on the posterior wall, in an area without plaque, with a clearly identified double line pattern. When atherosclerotic plaque was observed (thickness over 1.5 mm), its morphology was assessed with B-mode imaging, and in the next stage, the surface of each plaque was assessed—the blood flow spectrum was analyzed at the level of atherosclerotic plaques using the spectral recording option. In color imaging, the presence of acceleration typical of significant vasoconstriction was either excluded or confirmed. The carotid arteries on the opposite side were examined in an analogous manner. The mean value of the measurements for the right and left sides was taken as the final intima media thickness.

For comparative analysis, the study group of patients was divided using serum selenium concentrations and serum antioxidant status as median cut-off points. Based on the Se-S median of 88.48 μg/L, the subgroups of low-Se (Se-S < median) and high-Se (Se-S ≥ median) patients were distinguished. Based on the TAS median of 1.18 mM, the subgroups of low-TAS (TAS < median) and high-TAS (TAS ≥ median) patients were distinguished. For a similar purpose, the group of respondents was divided according to the cIMT median of 0.70 mm. On this basis, the low-cIMT (cIMT < median) and high-cIMT (cIMT ≥ median) patient subgroups were distinguished. Each group consisted of 38 patients.

The obtained results were statistically analyzed with the use of the “Dell Statistica 13” statistical package (Dell Inc., Tulsa, OK, USA). Results for quantitative variables were expressed as mean values ± standard deviations. The distribution of quantitative variables was checked using the Shapiro–Wilk test. For independent quantitative variables with a normal distribution, the *t*-test was used to test the significance of the hypotheses. In the case of variables with a distribution other than normal, the Mann–Whitney *U*-test was used for independent quantitative variables. Results for qualitative variables were expressed as percentages. The chi-square test was used to test the significance of the hypotheses for independent qualitative variables. To determine the correlations between the studied variables, correlation, and regression analysis was performed. The parameters of the model obtained in the regression analysis were estimated using the least squares method. The level of statistical significance was set at *p* < 0.05.

## 3. Results

Se-S and TAS in the entire group of patients suffering from arterial hypertension were 89.73 ± 18.99 µg/L and 1.18 ± 0.35 mM. Ultrasound exam of the arteries revealed the presence of atherosclerotic plaques in 59.2% of the patients. In the studied group of patients, the presence of insignificant carotid stenosis was observed in 14.5% of the patients. Significant carotid stenosis or carotid occlusion were not demonstrated. The cIMT measured by ultrasound was 0.68 ± 0.15 mm.

In comparative analyses, cIMT was significantly greater in patients with arterial hypertension with Se-S < median in comparison to patients with arterial hypertension with Se-S ≥ median, as well as in patients with arterial hypertension with the TAS < median than in patients with arterial hypertension with the TAS ≥ median. Se-S was statistically significantly lower in patients with arterial hypertension with the cIMT ≥ median compared to patients with arterial hypertension with the cIMT < median. The subgroups that differed in Se-S and TAS did not differ in the incidence of atherosclerotic plaques and carotid vasoconstriction. The full results of serum selenium concentrations (Se-S), total antioxidant status (TAS), and parameters of carotid ultrasound exam in the studied subgroups of patients are presented in Table 2.

**Figure 1:**
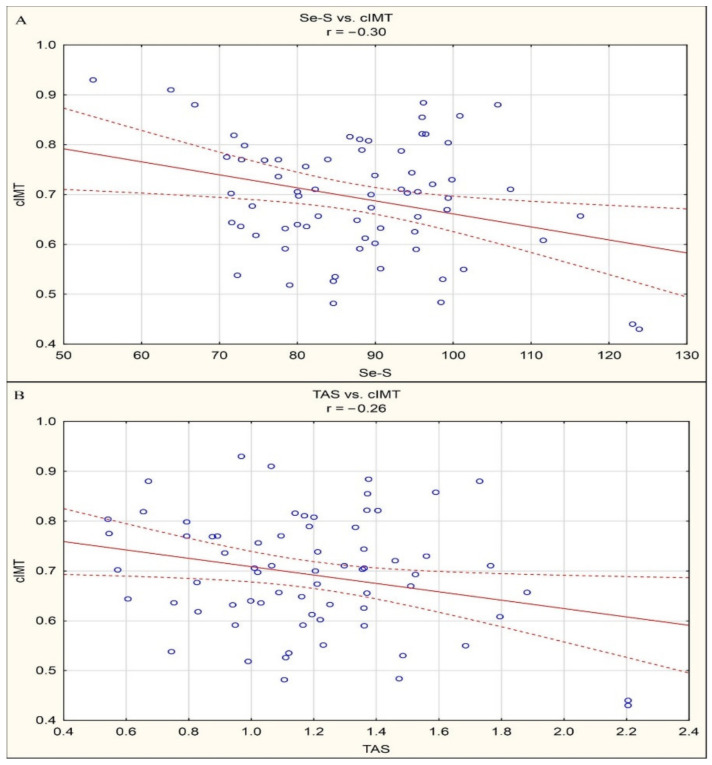
(**a**) Statistically significant correlations between Se-S and cIMT (r = −0.30, *p* < 0.05). (**b**) Statistically significant correlations between TAS and cIMT (r = −0.26, *p* < 0.05).

By means of correlation analysis, negative linear correlations between Se-S and cIMT (r = −0.30, *p* < 0.05) and between TAS and cIMT (r = −0.26, *p* < 0.05) were documented in the study group of patients. Statistically significant correlations are presented in Figure 1.

In the first stage of regression analysis, based on the univariate analysis, variables potentially related to the cIMT measured by ultrasound were selected. The analysis covered univariate regression models between the cIMT considered as the dependent variable and other variables considered independent, that is, basic anthropometric parameters (age, gender, BMI), smoking, taking antihypertensive drugs (diuretics, β-blockers, ACE inhibitors, angiotensin receptor blockers, calcium channel blockers), parameters of lipid metabolism (blood total cholesterol concentration, blood HDL cholesterol concentration, blood triglycerides concentration), the parameter of carbohydrate metabolism (blood glucose concentration), serum selenium concentrations, and total antioxidant status. Then, recognizing statistically significant correlations from univariate regression models as a method indicating potentially independent variables, using multivariate regression analysis, a regression model was obtained, defining the factors independently related to the cIMT in the study group of patients:

cIMT = 0.817 + 0.003 age + 0.004 BMI + 0.100 smoking − 0.004 Se-S ± 0.101.

The obtained regression model indicates that in the study group of patients, older age, higher BMI, smoking, and lower serum selenium concentrations are independent risk factors for the greater cIMT. The results of the estimation of variables related to the cIMT measured by ultrasound are presented in Table 3.

Since the correlation between Se-S and cIMT and TAS and cIMT was statistically significant in univariate regression analysis, and the correlation between Se-S and cIMT was statistically significant in multivariate regression analysis, with no statistical significance of the correlation between TAS and cIMT, the next step involved regression analysis concerning the determination of variables potentially related to TAS. Following the methodological procedure analogous to the estimation of cIMT-related variables, the following final regression model was obtained for TAS:

TAS = 0.191 − 0.116 BMI − 0.170 smoking + 0.109 ACE inhibitors + 0.138 β-blockers + 0.012 Se-S ± 0.266.

The obtained model shows that among the patients, higher BMI and smoking are independent risk factors for the lower TAS, and the use of ACE inhibitors, β-blockers, and higher Se-S are independent factors of protection against the lower TAS. The results of the estimation of variables related to the total antioxidant status are presented in Table 4.

## 4. Discussion

The obtained results indicate that patients with arterial hypertension with lower serum selenium concentrations and lower serum total antioxidant status are characterized by a greater carotid intima media thickness assessed by ultrasound. In comparative analyses, it was documented that cIMT was significantly greater in patients with hypertension with Se-S < median in comparison to patients with arterial hypertension with Se-S ≥ median, as well as in patients with arterial hypertension with TAS < median than in patients with arterial hypertension with the TAS ≥ median. Moreover, Se-S were statistically significantly lower in patients with arterial hypertension with the cIMT ≥ median compared to patients with arterial hypertension with the cIMT < median. In addition, the existence of a correlation between serum selenium concentrations and cIMT, as well as between serum selenium concentrations and serum total antioxidant status, was demonstrated by correlation analysis and univariate regression analysis.

Based on multivariate regression analysis, it was documented that while lower serum selenium concentrations are a statistically independent risk factor for increased ultrasound-assessed cIMT, the correlation of lower total antioxidant status and higher cIMT should be considered secondary to the relationship between Se-S and TAS. A significant correlation between Se-S and cIMT in the multivariate estimation was documented in the regression analysis, with no significance of the correlation between TAS and cIMT and the significance of the correlation between Se-S and TAS in an analogous type of estimation.

The obtained results are in line with the long-standing trend of investigating the relationship between the blood selenium concentration and the severity of early atherosclerotic lesions in the carotid arteries. The data that we have at our disposal based on previous research on this topic is inconclusive, moreover, it concerns various population groups. In the 1990s, Salonen et al. (1991 and 1993) indicated the existence of inversely proportional correlation between serum selenium concentrations and cIMT. In the first study conducted by this team, a group of 126 randomly selected Finns was examined and it was shown that the mean increase in maximum cIMT after two years was significantly greater in men with lower serum selenium concentrations compared to men with higher serum selenium concentrations (0.15 mm vs. 0.09 mm, with 1.40 mumol/L as the criterion separating the groups). At the same time, it was indicated that the above correlation was independent of age and tobacco smoking; moreover, attention was drawn to the synergy of higher serum copper levels, lower serum selenium concentrations, and higher LDL cholesterol in the process of atherogenesis [21]. Another study by the same team of researchers documented a similar correlation in patients with acute myocardial infarction. It has been shown that older age, higher serum LDL cholesterol, smoking, more intense platelet aggregation, higher serum copper levels, lower serum selenium concentrations, and higher blood hemoglobin levels are the strongest predictors of a two-year increase in IMT in the common carotid artery in patients in the acute myocardial infarction registry [22]. At the same time, however, we have the results of studies based on which the existence of a correlation between selenium concentrations in the body and cIMT was negated. An EVA study conducted on a group of 1187 men and women with no history of coronary artery disease and stroke regarding the correlation between antioxidant levels and cIMT indicated that vitamin E levels in erythrocytes were significantly negatively correlated with cIMT after considering conventional cardiovascular risk factors; however, no correlation was observed between selenium concentrations and carotenoids in plasma and cIMT. It should be noted, however, that the same study proved that with plasma selenium concentrations of <1 quartile, the unfavorable correlation between lipid peroxidation and the presence of atherosclerotic plaques was clearly marked in the cervical arteries [23]. Supplementary results to the importance of vitamin E were obtained in the Nutrition for Healthy Living study. In a group of 298 respondents, it was documented that elevated serum vitamin E levels are associated with abnormal markers of atherosclerosis and therefore may even increase the risk of cardiovascular complications in HIV-infected adults. In this study, no correlation was observed between serum selenium concentrations and cIMT [24]. After considering potential confounding factors, no significant correlation was observed between selenium concentrations and cIMT also in the studies conducted by Xun et al. It should be noted, however, that in this project a population of young adults was studied (3112 Americans aged 20–32), and selenium concentrations were measured in the toenails using instrumental neutron-activation analysis [25]. In studies on the discussed correlation between blood selenium concentrations and cIMT, there are also sporadic reports of a positive direction of this correlation. Swart et al. studied 987 South African adults and found a positive correlation between blood selenium concentrations and cIMT, especially in people with high serum selenium concentrations [26]. It should be emphasized that the results of our studies constitute an attempt to analyze the discussed correlation in a group of people different than people studied so far, namely patients with arterial hypertension without other clinically evident diseases of the cardiovascular system. When analyzing the results of the current study together with the studies conducted by other authors, it seems reasonable to say that the significant inversely proportional correlation between serum selenium concentrations and cIMT in our study was observable.

Some of the studies published so far on the importance of selenium for the development of early atherosclerotic lesions in the arteries differ significantly in terms of methodology. The authors of these studies use the supply of selenium in the diet as an indicator of selenium availability for the organism instead of selenium concentrations in biological material. For example, in the study conducted by Ferreira et al., the influence of antioxidant consumption on the occurrence of subclinical cardiovascular disease (CVD) was analyzed in a group of 96 postmenopausal women. Food consumption was assessed using a validated food frequency questionnaire. Subclinical CVD was defined as cIMT > 0.9 mm and/or the presence of at least one plaque in any of the segments of the cervical arteries examined. It has been shown, among others, that study participants with subclinical CVD significantly more often consumed lower doses of selenium in their diet [27]. The Kardiovize study assessed the correlation between the composite dietary antioxidant index (CDAI) based on zinc, selenium, vitamin A, vitamin C, vitamin E, and carotenoid intake and cIMT in a large group of 894 subjects. It was shown that lower CDAI, along with older age, higher systolic blood pressure, and higher triglyceride levels was a significant predictor of an increase in cIMT in women. There was no similar correlation between the CDAI and cIMT in men. Maugeri et al. conclude that the combined intake of antioxidant nutrients may prevent the initiation and progression of arterial lesions, but in a gender-dependent manner [28].

The correlation between Se-S and cIMT has prompted some researchers to consider selenium supplementation as a potential non-pharmacological method of reducing cardiovascular risk. The SU.VI.MAX study contradicts the effectiveness of such supplementation. Zureik et al. showed that people receiving a combination of antioxidants in low doses (120 mg of vitamin C, 30 mg of vitamin E, 6 mg of beta carotene, 100 micrograms of selenium, and 20 mg of zinc) daily compared to those receiving placebo, after a follow-up period of about seven years did not differ in cIMT assessed via ultrasound (0.70 ± 0.08 vs. 0.70 ± 0.08 mm, *p* > 0.05) [29]. Interesting results in this context have recently been provided by the study conducted by Liberda et al., which assessed the body’s load with environmental pollutants and its correlation with cIMT. This study included 535 indigenous people from the Eeyou Istchee territory of Canada, who had a higher burden of cardiovascular morbidity and mortality than non-native people in Canada. It has been shown that selenium compounds, which are a component of environmental pollutants, can, together with nickel and cadmium compounds, although to a lesser extent, be responsible for an increase in cIMT, which is a marker of cardiovascular risks [30]. In the context of the above, it can be concluded that there is a statistical relationship between blood selenium concentration and cIMT thickness. However, the existence of this relationship does not authorize selenium supplementation as a method of reducing cardiovascular risk.

The results of our studies indicate a lower total antioxidant status as an indirect indicator of early atherosclerotic lesions in the carotid arteries, conditioned by low blood selenium concentrations. Thus, they constitute an important voice in the discussion on the correlation between the total antioxidant status and cIMT, in which, in recent studies, there are reports both confirming the existence of one and excluding it. Similar results to those currently discussed were obtained by Skoczynska et al. and Bogdanski et al. [31,32]. The first of these studies involved 154 employees of a chemical plant that used mercury to produce chlorine. A negative linear correlation has been documented between the serum total antioxidant status and cIMT (r = −0.21, *p* < 0.05). It was concluded that occupational exposure to mercury accelerates the occurrence of early carotid atherosclerotic lesions, and the disturbance of the antioxidant status of the body may be responsible for their development [31]. The second of these studies was performed with the participation of patients newly diagnosed with essential hypertension (*n* = 42) and a control group of healthy participants (*n* = 20). It showed that cIMT was significantly greater, while the TAS was significantly lower in patients with hypertension compared to the control group. At the same time, however, multivariate regression analysis, like in our studies, did not show that the TAS was an independent predictor of an increase in cIMT [32]. The results confirming the existence of a correlation between TAS and IMT, confirmed by regression analysis, were obtained in the studies by Gur et al. (2007, 2014) [33,34]. It should be noted, however, that IMT in both studies was measured in the thoracic aorta during transesophageal echocardiography (TEE) performed for a variety of typical clinical indications. In a study published in 2007, 70 patients were examined, documenting an independent correlation between greater IMT of the thoracic aorta with the lower TAS, as well as with more extensive damage to lymphocyte DNA, higher total cholesterol, and higher LDL cholesterol in multiple linear regression analysis [33]. In a study conducted in 2014 on a group of 133 patients, it was shown by multiple linear regression analysis that greater IMT of the thoracic aorta was independently correlated with lower TAS, as well as with lower PON1 paraoxonase activity, older age, and higher LDL cholesterol levels [34]. As examples of studies that did not confirm the correlation between the TAS and cIMT, the publications of Tabatabaei et al. and Cakir et al. can be listed [35,36]. Among 267 patients included in the study conducted by Tabatabaei et al. significantly lower values of the TAS and greater cIMT were observed in diabetic patients than in non-diabetic patients. At the same time, however, no direct correlation between the TAS and cIMT was demonstrated [35]. Cakir et al. examined 52 patients with polycystic ovary syndrome and 36 people in the control group, who matched in terms of age and body mass index. It was shown that although cIMT was greater in patients with polycyclic ovary syndrome than in the control group, the TAS marker was similar in both groups. There was no correlation between cIMT and TAS [36]. Similarly, no significant correlation was found between the TAS and other parameters of oxidative/antioxidant status and cIMT in the study conducted by Karkucak et al. [37]. However, this study assessed the population of patients with ankylosing spondylitis (47 patients), paying attention to the importance of treatment with a tumor necrosis factor (anti-TNF) inhibitor (23 patients). Summing up, the currently discussed study can be treated as a verification of the previously published ambiguous results of studies conducted in populations of people suffering from other diseases. It points to the existence of a correlation between the TAS and cIMT as secondary to the correlation between Se-S and TAS in an independent correlation between Se-S and cIMT.

A potential pathogenic mechanism of atherosclerosis acceleration because of selenium deficiency is the shift of the redox balance of the organism, because of the reduction of the antioxidant potential, towards increased oxidative activity. Selenium deficiency, due to selenoprotein deficiency, reduces the effectiveness of removing lipid oxidation products, mainly lipid hydroperoxides. Together with other oxidative mechanisms, it contributes to damaged endothelium, causes inflammation, increased blood prothrombotic activity, spasms, and permanent/irreversible proliferation of vascular walls.

The discussed studies have several major limitations. In terms of the study group, a significant limitation is its limited number, overrepresentation of overweight or obese patients, and small number of patients with selenium deficiency. In terms of methodology, the main limitation of the study is the lack of determination of selenoproteins and markers of inflammation. In light of current research, it is believed that the selenoprotein P concentration in plasma is the most conclusive marker for determining the optimum supply of selenium [38]. In addition, if inflammation is present, Se status measured in plasma/serum decreases because of reduced expression of selenoprotein P, the major component of Se in plasma [39]. Moreover, methodological limitations must be considered, including the lack of objective measurements of current blood pressure values (e.g., ambulatory blood pressure monitoring, ABPM), no determination of the total oxidative status, and no long-term cIMT assessment.

## 5. Conclusions

In patients with arterial hypertension, the lower total antioxidant status conditioned by lower serum selenium concentrations may be correlated with an increase in the carotid intima media thickness assessed by ultrasound.

## Figures and Tables

**Table 1:** Clinical characteristics of the studied group of patients.

Age (Years) ^a^	53.48 ± 12.78
men ^b^	60.5 (46)
women ^b^	39.5 (30)
height (cm) ^a^	172.76 ± 10.93
body mass (kg) ^a^	94.99 ± 18.22
BMI (kg/m^2^) ^a^	31.70 ± 4.69
normal body mass ^b^	3.9 (3)
overweight ^b^	38.1 (29)
obese ^b^	57.9 (44)
smoking ^b^	21.0 (16)
arterial hypertension ^b^	100.0 (76)
diuretics ^b^	50.0 (38)
β-blockers ^b^	51.3 (39)
ACE inhibitors ^b^	35.5 (27)
angiotensin receptor blockers ^b^	40.8 (31)
calcium channel blockers ^b^	51.3 (39)
total cholesterol (mg/dL) ^a^	205.36 ± 44.57
HDL cholesterol (mg/dL) ^a^	48.79 ± 13.44
LDL cholesterol (mg/dL) ^a^	121.46 ± 36.05
triglicerides (mg/dL) ^a^	198.95 ± 145.44
glucose (mg/dL) ^a^	100.05 ± 22.07

^a^ quantitative variable expressed as mean ± standard deviation; ^b^ qualitative variable expressed as a percentage (number); ACE—angiotensin converting enzyme; BMI—body mass index; HDL—high-density lipoprotein; LDL—low-density lipoprotein.

**Table 2:** Serum selenium concentration (Se-S), total antioxidant status (TAS), and parameters of ultrasound examination of carotid arteries in the studied subgroups of patients.

Subgroup	Se-S (µg/L) ^a^	TAS (mM) ^a^	Atherosclerotic Plaques ^b^	Non-Significant Stenosis ^b^	cIMT (mm) ^a^
low-Se	76.86 ± 7.89	0.98 ± 0.22	63.2	15.8	0.73 ± 0.19
high-Se	102.61 ± 18.11	1.39 ± 0.34	55.3	13.1	0.65 ± 0.10
*p*	<0.05	<0.05	Ns	Ns	<0.05
low-TAS	83.94 ± 0.91	0.91 ± 0.19	63.2	18.4	0.79 ± 0.18
high-TAS	95.53 ± 12.18	1.45 ± 0.25	55.3	10.5	0.56 ± 0.13
*p*	<0.05	<0.05	Ns	Ns	<0.05
low-cIMT	94.98 ± 22.26	1.25 ± 0.38	21.0	0.0	0.57 ± 0.05
high-cIMT	84.49 ± 13.37	1.13 ± 0.33	97.4	28.9	0.80 ± 0.12
*p*	<0.05	ns	<0.05	<0.05	<0.05

^a^ quantitative variable expressed as mean ± standard deviation; ^b^ qualitative variable expressed as a percentage (number); cIMT—carotid intima media thickness; Se-S—serum selenium concentration; TAS—total antioxidant status.

**Table 3:** Results of regression analysis in the studied group of patients with arterial hypertension: model estimation for the dependent variable cIMT (mm).

	Model for: cIMT (mm)					
	Univariate Regression			Multivariable Regression		
	Rc	SEM of Rc	*p*	Rc	SEM of Rc	*p*
age (years)	0.003	0.001	<0.05	0.003	0.001	<0.05
men	0.070	0.031	<0.05	0.060	0.036	ns
BMI (kg/m^2^)	0.003	0.001	<0.05	0.004	0.001	<0.05
smoking	0.123	0.008	<0.05	0.100	0.032	<0.05
diuretics	−0.034	0.031	Ns	-	-	-
β-blockers	−0.022	0.003	<0.05	−0.009	0.011	ns
ACE inhibitors	−0.016	0.033	Ns	-	-	-
angiotensin receptor blockers	−0.039	0.032	Ns	-	-	-
calcium channel blockers	−0.023	0.003	<0.05	−0.032	0.024	ns
total cholesterol (mg/dL)	0.001	0.001	Ns	-	-	-
HDL cholesterol (mg/dL)	−0.001	0.001	Ns	-	-	-
triglicerides (mg/dL)	0.001	0.000	<0.05	0.001	0.001	ns
glucose (mg/dL)	0.001	0.001	Ns	-	-	-
Se-S (μg/L)	−0.004	0.001	<0.05	−0.005	0.001	<0.05
TAS (mM)	−0.184	0.045	<0.05	−0.116	0.087	ns

ACE—angiotensin-converting enzyme; BMI—body mass index; cIMT—carotid intima media thickness; HDL—high-density lipoprotein; Rc—regression coefficient; SEM—standard error of mean; Se-S—serum selenium concentration; TAS—total antioxidant status.

**Table 4:** Results of regression analysis in the studied group of patients with arterial hypertension: model estimation for the dependent variable TAS (mM).

	Model for: TAS (mM)					
	Univariate Regression			Multivariable Regression		
	Rc	SEM of Rc	*p*	Rc	SEM of Rc	*p*
age (years)	−0.003	0.003	Ns	-	-	-
men	0.116	0.079	Ns	-	-	-
BMI (kg/m^2^)	−0.012	0.002	<0.05	−0.016	0.007	<0.05
smoking	−0.227	0.092	<0.05	−0.170	0.085	<0.05
diuretics	0.131	0.057	<0.05	0.048	0.068	ns
β-blockers	0.117	0.008	<0.05	0.138	0.026	<0.05
ACE inhibitors	0.181	0.032	<0.05	0.109	0.040	<0.05
angiotensin receptor blockers	0.081	0.079	ns	-	-	-
calcium channel blockers	0.019	0.078	ns	-	-	-
total cholesterol (mg/dL)	−0.001	0.001	ns	-	-	-
HDL cholesterol (mg/dL)	0.002	0.003	ns	-	-	-
triglicerides (mg/dL)	−0.001	0.001	ns	-	-	-
glucose (mg/dL)	−0.002	0.002	ns	-	-	-
Se-S (μg/L)	0.010	0.002	<0.05	0.012	0.002	<0.05

ACE—angiotensin-converting enzyme; BMI—body mass index; cIMT—carotid intima media thickness; HDL—high-density lipoprotein; Rc—regression coefficient; SEM—standard error of mean; Se-S—serum selenium concentration; TAS—total antioxidant status.

## Data Availability

The data presented in this study are available upon request from the corresponding author. The data are not publicly available.
